# A systematic analysis for disease burden, risk factors, and trend projection of Alzheimer’s disease and other dementias in China and globally

**DOI:** 10.1371/journal.pone.0322574

**Published:** 2025-05-07

**Authors:** Siyu Liu, Daoying Geng

**Affiliations:** 1 Radiology Department, Huashan Hospital, Affiliated with Fudan University, Shanghai, China; 2 Shanghai Engineering Research Center of Intelligent Imaging for Critical Brain Diseases, Shanghai, China; 3 Institute of Functional and Molecular Medical Imaging, Fudan University, Shanghai, China; National center for chronic and non-communicable diesease prevention and control, CHINA

## Abstract

**Background:**

This study aims to provide improvement directions for aging societies by analyzing the disease burden, risk factors and trend forecasts of AD and other dementias (ADD) in China and globally from 1990 to 2021.

**Methods:**

Data sourced from Global Burden of Disease 2021. We extracted indicators of disease burden and risk factors for ADD in people aged 40 years and older, including incidence, prevalence, deaths, disability-adjusted life years, years lived with disability and years of life lost. The annual percent change and average annual percent change over the past 32 years were analyzed by Joinpoint regression. Decomposition analysis was used to clarify the contribution of aging, population and epidemiological change. The directions of deaths and incidence in China and globally were predicted using ARIMA model for the next 15 years.

**Results:**

The disease burden of ADD in China is heavier than in most countries and regions. By 2021, China’s disease burden has increased by three times, while the global disease burden has doubled. Females bear more burden but face lower mortality. Population growth is the main reason for the burden. Smoking, high fasting plasma glucose and high body-mass index are the three major risk factors, among which high fasting plasma glucose occupies a dominant position.

**Conclusion:**

The disease burden of ADD in China and globally is increasing daily and will remain high in the future. It is urgent to introduce some effective intervention measures to prevent such diseases as early as possible.

## Introduction

Alzheimer’s disease and other dementias (ADD) are a category of primary degenerative diseases of the central nervous system, which have become an increasingly serious global health issue with no specific countermeasures available yet [1]. The clinical manifestations of ADD often include memory impairment, changes in personality and behavior, progressive loss of ability to perform daily activities, ultimately leading to disability or death among the elderly [2,3]. ADD are also the fifth leading cause of death worldwide [1]. Studies have shown that by 2050, 152 million people worldwide will be affected by ADD, and the global economic cost will amount to $9.12 trillion [1,4]. It is also worth noting that families and caregivers of ADD patients take on more mental stress and negative emotions, so the social and familial burdens of caring for the ADD population are significant and unsustainable [5]. This issue is also one that cannot be ignored in ADD and does not yet have an optimal solution.

There have been some researches on the trends of global ADD changes, including the analysis of gender, age, and regional distribution of ADD, as well as the exploration of risk factors for dementias, mostly up to the year 2019 [6–8]. Globally, there are numerous attributable risk factors for ADD, including aging, hypertension, obesity, diabetes, smoking, depression, lack of physical activity, low educational attainment, infrequent social interaction, excessive alcohol consumption, brain injury, and air pollution [9,10]. This indicates that it is feasible to develop targeted preventive measures to alleviate the economic burden and mental stress. Moreover, studies have already shown that controlling risk factors can effectively reduce the incidence of ADD [10,11]. As one of the countries with a more severe aging population, China had over 13 million patients with ADD in 2019, and the number is projected to reach 115 million by 2050. However, there is currently limited precise understanding of the disease burden and risk factors of ADD specifically in China, which also hinders the identification of the specific causes of ADD in the country [12]. The consequence of not being able to target and reduce the incidence of ADD in China will be that the global ADD burden remains high, or even continues to rise.

In the field of epidemiological research, the Global Burden of Disease (GBD) is a commonly used free public database jointly initiated by institutions such as the World Bank and the World Health Organization. The data in the GBD database are aggregated over multiple years for each country globally, with regions as the unit of analysis. Its data coverage is extensive, encompassing all major diseases and injuries, corresponding risk factors, and a full range of geographical and demographic information. As of now, GBD 2021 contains data on 371 diseases and injuries and the corresponding major 88 risk factors across 204 countries and regions. In October 2020, GBD 2019 was published in “*The Lancet*” [13]. Currently, almost all published articles regarding the situation of ADD in China or globally utilize data from GBD 2019. While, in July 2023, “*Lancet*” published GBD 2021, which included new data for the years 2020 and 2021 for all indicators, and also incorporated new risk factors [14]. The new data is conducive to identifying new health issues and high-risk populations, allowing for the formulation of more targeted intervention measures.

Therefore, this research included the disease burden data and new attributable risk factors of ADD in China and globally from 1990 to 2021 from the GBD 2021 database. Our objective is to analyze these data to help formulate effective preventive measures as soon as possible, address and optimize the allocation of health resources to some extent, and promote in-depth research and understanding of public health issues.

## Materials and methods

### Study overview and data sources

This study examines the burden of disease, attributable risk factors, and trend analysis of ADD in China and globally from 1990 to 2021, with the aim of realizing early interventions to alleviate the economic burden on society and mental stress on individuals. All data were derived from publicly available datasets in the Global Health Data Exchange (GHDx) (https://ghdx.healthdata.org/). We used “GBD results” and “GBD compare” to view and download risk factors and epidemiological and burden of disease assessments of ADD in China and the world from 1990 to 2021 (https://vizhub.healthdata.org/gbd-results/, May 16, 2024). ADD is more common in people aged 40 and above, so this research focused on screening and analyzing the burden of disease in people aged 40 years and above. Meanwhile, all mentions of “all-age numbers” in this study refer to individuals aged 40 and above. We extracted detailed indicators of the burden of disease, including incidence, prevalence, deaths, disability-adjusted life years (DALYs), years lived with disability (YLDs) and years of life lost (YLLs), and organized the data for subsequent statistical analyses.

### Definitions of disease and measures

Dementia is a clinical syndrome due to neurodegenerative changes, which is characterized by cognitive decline, memory loss, and difficulty in thinking skills, and in severe cases affects daily life [15,16]. The main forms of dementia include AD, vascular dementia, Lewy body dementia, and frontotemporal lobe dementia, of which AD is the most common, but a mixture of the different forms of dementia coexist as well [12,16]. For the GBD analysis, our study adopted a definition of dementia based on the International Classification of Diseases, 10^th^ Revision (ICD-10), which covered the six disease type codes it identifies, including F00, F01, F02, F03, G30, and G31 [17,18].

This study collected the data of the all-age numbers and the age-standardized rates per 100,000 persons of incidence, prevalence, deaths, DALYs, YLDs, and YLLs for ADD. Incidence represents the number of occurrences of the disease during this year, while prevalence is the cumulative annual number of occurrences of this disease in the current year. Deaths is the number of deaths from the disease in the current year. All three are population-based estimates, and their rates per 100,000 are age-standardized incidence rate (ASIR), age-standardized prevalence rate (ASPR), and age-standardized death rate (ASDR), respectively.

DALYs refers to the level of disease burden, which can be viewed as one “healthy” year lost due to disability or death from some cause. YLDs is measured by multiplying the prevalence of a disease by the disability weight of that disease. The disease weights reflect the severity of the different conditions, which are derived from surveys of the public. YLLs indicates the number of years lost due to premature death, which is calculated by subtracting the age at death from the maximum possible life expectancy of a person at that age. DALYs is obtained by adding YLDs and YLLs.

### Statistical analysis

In this research, the statistical description comprehensively analyzes all disease burden indicators, demonstrating the corresponding values and 95% uncertainty intervals (95% UI). 95% UIs obtained from the 25th and 975th values of an ordered sample of 1000 cases. Due to the inconsistent age and gender structures, the disease burden indicators of ADD in China cannot be directly compared with those globally. Therefore, we need to calculate comparable rates using the same population structure, which are age-standardized rates (ASRs). The choice of “standard” often employs the population composition of a large sample group, such as that of a province, a country, or the world. All ASRs in this research were calculated using the 2021 GBD global population standard [19,20]. The ASRs with different standards used for standardization, but the relative relationships among the comparison results remain unchanged. The total percentage change between 1990 and 2021 was found by dividing the difference between the 2021 estimates and the 1990 estimates by the 1990 estimates [20].

Joinpoint regression model uses linear regression to fit the data and is used to analyze significant trend change points in time-series data, turning general trendiness into multiple local trendiness. Compared with commonly used linear models and time series models, the Joinpoint regression model does not impose strict requirements on whether the data sequence itself exhibits a trend. Instead, it is capable of identifying and quantifying Joinpoints within the data, thereby segmenting the data into different trend phases. The model fitting is accomplished by the Grid Search Method (GSM), and the model selection is determined by the Monte Carlo permutation test [21,22]. Joinpoint regression, developed by the National Cancer Institute, assesses whether trends are statistically significant by analyzing annual percent change (APC) and average annual percent change (AAPC) compared to 0 [23,24]. AAPC is the geometrically weighted average of the percentage changes over different years in joinpoint analysis, with the weights equal to the length of each period within the specified time interval [22]. We also calculated the corresponding 95% confidence interval (95% CI) for each AAPC from the linear regression model. When the AAPC and its 95% CI are greater than 0, the corresponding ASR is considered to show an increasing trend. While when the values are equal to or less than 0, the corresponding ASR shows a steady or decreasing trend [25].

Decomposition analysis is a method used to understand patterns and changes in data, which can help us comprehend how different factors influence the overall variation. The analysis uses the Das Gupta method to decompose the change in the number of ill people over a certain period of time into changes in population age structure, population growth, and epidemiologic trends. The Das Gupta method uses mathematical decomposition techniques to divide the overall change into several parts in order to determine the precise contribution of each component [26]. This study uses decomposition analysis to dissect the incidence and death of ADD from 1990 to 2021 in order to observe the impacts of changes in aging, population and epidemiological change on ADD. The first two represent the impact of aging and population growth on the burden of disease, while epidemiologic change refers to ASDR and ASIR after changes in underlying age and population [27].

Finally, we used autoregressive integrated moving average (ARIMA) model to predict ASDR and ASIR in China and globally in 2036 to clarify the future direction of the disease. The ARIMA model is a commonly used time series analysis method that effectively captures trends and seasonal variations in time series data by combining three main components: autoregression (AR), increment (I) and moving average (MA) [6,28]. The parameters of the model are usually denoted as ARIMA(p,d,q), where p and q represent the order of AR and MA, and d is the number of I [29]. “P” represents using the values from the previous “p” time points to predict the current value. “Q” denotes utilizing the previous “q” error terms to anticipate the current value. “D” means the number of incremental operations needed to make the data a stationary sequence. Subsequently, prediction of future data after fitting the best values of p, d, and q based on the Akaike information criterion (AIC) through the *auto.arima()* function and the *forecast()* function [30].

Joinpoint regression program (version 5.1.0) were used to conduct the joinpoint analysis. All remaining statistical analyses were done by the R software (version 4.3.3). The visualization of the results of the statistical description and the statistical analysis were both done by the package *“ggplot2”*. *P* values less than 0.05 were considered statistically significant differences.

## Results

### Distribution map of disease burden for ADD

**Fig 1:**
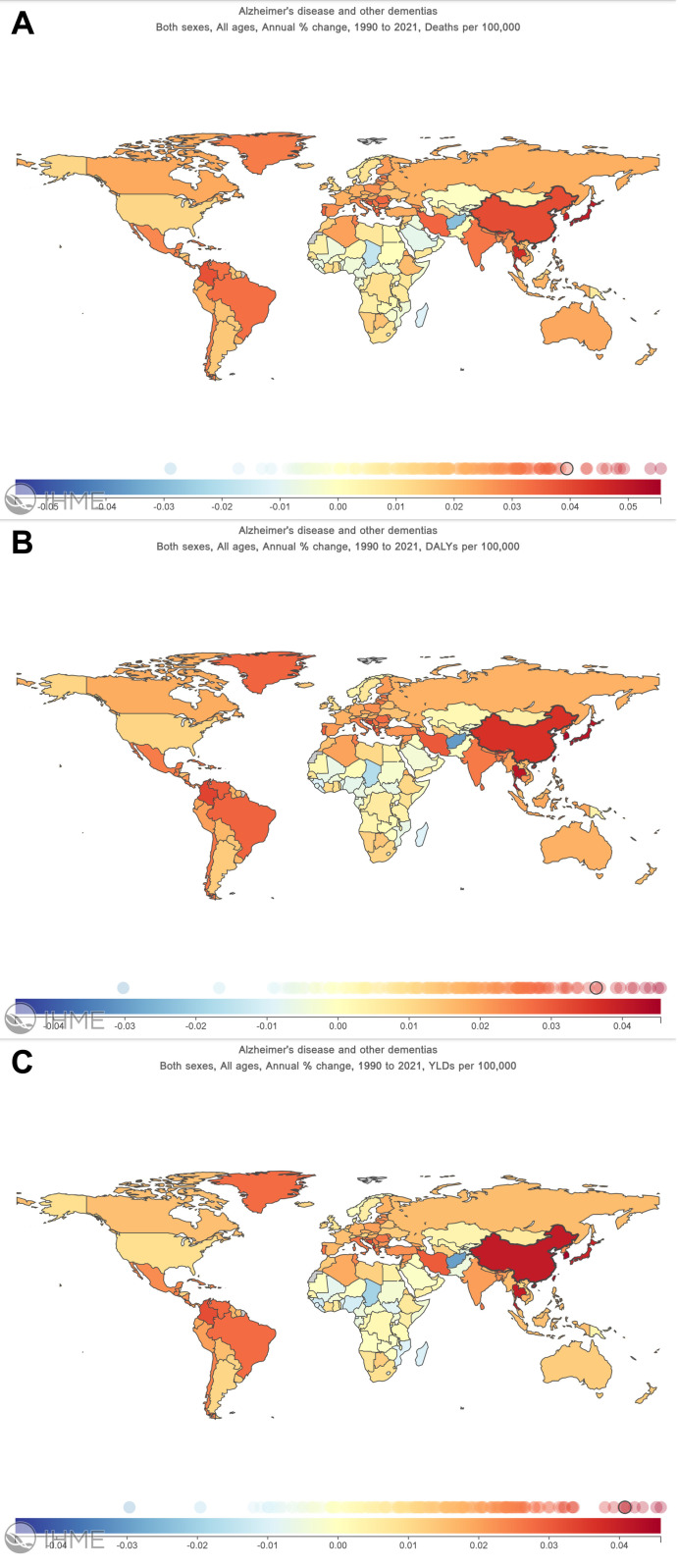
Global burden of disease map for ADD, 1990-2021. (A) Annual change in deaths per 100,000 people for all sexes and all ages (B) Annual change in DALYs per 100,000 people for all sexes and all ages (C) Annual change in YLDs per 100,000 people for all sexes and all ages.

From a global perspective, the burden of disease for ADD is steadily increasing for all genders as well as for all ages from 1990 to 2021. As shown in Fig 1, excluding Afghanistan and some African countries, annual changes in Deaths per 100,000, DALYs per 100,000, and YLDs per 100,000 of all remaining countries and regions are greater than zero. We found that China has one of the highest disease burdens of AD and other dementias in the world, with annual changes in Deaths per 100,000 of 3.94%, in DALYs per 100,000 of 3.63%, and YLDs per 100,000 of 4.08%.

### Overview

Tables 1 and 2 provided a clearer picture of the detailed changes in the burden of disease indicators for ADD in China and the world between 1990 and 2021. In China, the incidence, prevalence, deaths, and YLDs of ADD have increased by 3-fold, and DALYs and YLLs have increased by 2-fold. Over the past 32 years, all-age numbers of incidence, prevalence and deaths have increased from 703,178 (95% UI, 601,506–808,633) to 2,914,112 (95% UI, 2,504,728–3,350,743), from 4,024,536 (95% UI, 3,446,398–4,623,086) to 16,990,827 (95% UI, 14,488,494–19,672,741) and from 119,809 (95% UI, 28,349–322,103) to 491,774 (95% UI, 124,968–1,330,182) (Table 1). After removing the effects of population growth and aging, ASRs showed a slight upward trend over the same period, but total percentage changes of all genders and women’s ASDRs (both: -0.02%; 95% UI, -0.16% to 0.18%; female: -0.02%; 95% UI, -0.21% to 0.23%) and YLLs (both: -0.03%; 95% UI, -0.17% to 0.18%; female: -0.04%; 95% UI, -0.22% to 0.23%) show negative values (Table 1). Thus, the trend direction for males is contrary to that of females on these two indicators, but otherwise the burden of disease is higher for women than for men. Compared with the situation in China, the global burden of disease for ADD was rising more slowly, and the total percentage change in ASRs over the same period were closer to zero. ASIR, ASPR and ASDR increased by 0.02% (95% UI, 0.01% to 0.03%), 0.03% (95% UI, 0.02% to 0.04%) and 0% (95% UI, -0.04% to 0.07%) respectively (Table 2). Similarly, ASRs of DALYs, YLDs and YLLs raised by 0.01% (95% UI, -0.03% to 0.05%), 0.03% (95% UI, 0.01% to 0.04%) and 0.01% (95% UI, -0.04% to 0.07%) (Table 2). The values and trends in the burden of ADD in men and women are not significantly different worldwide.

**Table 1:** All-age numbers and ASR per 100,000 of incidence, prevalence, deaths, DALYs, YLDs and YLLs for ADD and total percentage change by sex in China, 1990-2021.

	1990		2021		Total percentage change (%) (95% UI), 1990–2021	
Measure	All-age numbers (95% UI)	Age-standardized rate per 100,000 (95% UI)	All-age numbers (95% UI)	Age-standardized rate per 100,000 (95% UI)	All-age numbers	Age-standardized rate per 100,000
**Incidence**						
both	703178 (601506,808633)	121.11 (105.5,137.99)	2914112 (2504728,3350743)	151.47 (131.22,173.34)	3.14 (2.98,3.32)	0.25 (0.21,0.28)
female	442528 (381567,507194)	135.42 (118.39,154.07)	1836815 (1593651,2101343)	171.81 (150.12,195.9)	3.15 (3,3.32)	0.27 (0.23,0.3)
male	260650 (222339,301788)	100.25 (86.5,115.16)	1077297 (908448,1248194)	126.48 (107.78,145.62)	3.13 (2.92,3.32)	0.26 (0.22,0.3)
**Prevalence**						
both	4024536 (3446398,4623086)	703.14 (608.36,809.51)	16990827 (14488494,19672741)	900.82 (770.92,1043.22)	3.22 (3.05,3.39)	0.28 (0.24,0.31)
female	2512934 (2165052,2892724)	785.19 (681.22,900.41)	10828630 (9315735,12515957)	1025.11 (879.04,1186.81)	3.31 (3.13,3.49)	0.31 (0.26,0.34)
male	1511602 (1280688,1737520)	574.55 (493.64,666.55)	6162198 (5142286,7141800)	731.21 (618.54,851.63)	3.08 (2.86,3.28)	0.27 (0.22,0.31)
**Deaths**						
both	119809 (28349,322103)	31.39 (7.6,83.63)	491774 (124968,1330182)	30.82 (7.88,82.43)	3.1 (2.48,4.04)	-0.02 (-0.16,0.18)
female	80212 (19176,212442)	34.61 (8.32,90.6)	328431 (83715,862460)	33.8 (8.6,87.19)	3.09 (2.26,4.28)	-0.02 (-0.21,0.23)
male	39597 (9247,113675)	25.12 (6,70.58)	163343 (40664,466660)	25.9 (6.51,73.2)	3.13 (2.23,4.28)	0.03 (-0.15,0.26)
**DALYs**						
both	2702484 (1239177,6085395)	534.47 (236.2,1190.6)	10072478 (4947154,22219154)	562.39 (271.16,1238.81)	2.73 (2.22,3.3)	0.05 (-0.09,0.22)
female	1729685 (790232,3750294)	596.71 (265.21,1288.3)	6500199 (3171765,13681029)	631.38 (305.95,1318.24)	2.76 (2.1,3.5)	0.06 (-0.13,0.26)
male	972799 (434085,2307848)	429.85 (186.23,998.45)	3572279 (1694716,8148478)	463.67 (214.26,1055.84)	2.67 (2.07,3.36)	0.08 (-0.07,0.27)
**YLDs**						
both	808456 (545684,1082808)	145.78 (99.22,193.23)	3460324 (2394267,4632167)	185.63 (127.98,246.72)	3.28 (3.08,3.47)	0.27 (0.24,0.3)
female	521766 (354162,698505)	166.61 (114.06,222.28)	2275550 (1555763,3049867)	216.38 (147.99,288.88)	3.36 (3.17,3.55)	0.3 (0.26,0.33)
male	286690 (194753,376878)	113.18 (77.45,149.5)	1184774 (818129,1591260)	143.7 (99.59,192.13)	3.13 (2.92,3.35)	0.27 (0.22,0.31)
**YLLs**						
both	1894028 (432458,5187996)	388.69 (93.2,1021.16)	6612154 (1661583,18403595)	376.75 (96.26,1033.14)	2.49 (1.93,3.27)	-0.03 (-0.17,0.18)
female	1207919 (280413,3189050)	430.1 (103.45,1125.07)	4224649 (1064649,11432702)	415 (105.04,1115.53)	2.5 (1.74,3.56)	-0.04 (-0.22,0.23)
male	686109 (161864,2019592)	316.67 (74.59,872.59)	2387505 (592422,7003086)	319.98 (79.26,898.94)	2.48 (1.66,3.51)	0.01 (-0.19,0.27)

**Table 2:** All-age numbers and ASR per 100,000 of incidence, prevalence, deaths, DALYs, YLDs and YLLs for ADD and total percentage change by sex globally, 1990-2021.

	1990		2021		Total percentage change (%) (95% UI), 1990–2021	
Measure	All-age numbers (95% UI)	Age-standardized rate per 100,000 (95% UI)	All-age numbers (95% UI)	Age-standardized rate per 100,000 (95% UI)	All-age numbers	Age-standardized rate per 100,000
**Incidence**						
both	3834526 (3367544,4358428)	116.97 (102.77,132.32)	9837056 (8620519,11163700)	119.76 (104.96,135.89)	1.57 (1.51,1.62)	0.02 (0.01,0.03)
female	2482207 (2183968,2820920)	127.82 (112.82,144.18)	6191564 (5432752,7009226)	132.29 (116.3,149.8)	1.49 (1.44,1.55)	0.03 (0.02,0.04)
male	1352319 (1177680,1551793)	100.69 (88.05,114.43)	3645492 (3144738,4183541)	103.4 (89.45,118.45)	1.7 (1.63,1.75)	0.03 (0.01,0.04)
**Prevalence**						
both	21799761 (19067087,24837693)	672.22 (588.73,763.95)	56856688 (49382064,64977512)	694.01 (602.88,794.08)	1.61 (1.56,1.66)	0.03 (0.02,0.04)
female	14143446 (12361842,16105292)	736.15 (646.05,834.27)	36103380 (31468185,41117470)	769.94 (670.71,877.57)	1.55 (1.51,1.6)	0.05 (0.03,0.06)
male	7656315 (6611276,8728099)	571.47 (497.63,654.31)	20753308 (17769420,23796798)	589.47 (507.48,678.79)	1.71 (1.65,1.77)	0.03 (0.01,0.04)
**Deaths**						
both	663294 (163580,1764991)	25.04 (6.29,66.28)	1952677 (512981,4984737)	25.16 (6.68,64.25)	1.94 (1.77,2.2)	0 (-0.04,0.07)
female	462912 (115213,1215530)	27.6 (6.96,71.87)	1325805 (356478,3316451)	27.88 (7.48,69.79)	1.86 (1.66,2.15)	0.01 (-0.05,0.09)
male	200383 (47757,550521)	20.18 (4.98,55.7)	626872 (153869,1677847)	20.71 (5.19,55.5)	2.13 (1.94,2.41)	0.03 (-0.02,0.09)
**DALYs**						
both	13572308 (6439341,29586865)	445.75 (206.08,958.03)	36332687 (17237624,76873276)	450.98 (212.69,950.16)	1.68 (1.56,1.8)	0.01 (-0.03,0.05)
female	9106751 (4330350,19615098)	495.05 (231.43,1054.27)	23808560 (11368143,49746524)	504.87 (241.04,1055.02)	1.61 (1.47,1.76)	0.02 (-0.03,0.07)
male	4465557 (2092517,9971768)	362.99 (164.92,799.27)	12524126 (5871758,27158683)	372.53 (170.89,805.03)	1.8 (1.68,1.97)	0.03 (-0.02,0.08)
**YLDs**						
both	4409783 (3029439,5820654)	138.31 (94.96,182.44)	11582108 (7961942,15296793)	141.95 (97.72,187.2)	1.63 (1.57,1.68)	0.03 (0.01,0.04)
female	2959271 (2024970,3942682)	155.6 (106.59,206.6)	7602654 (5202893,10072814)	161.92 (110.67,214.71)	1.57 (1.52,1.62)	0.04 (0.03,0.05)
male	1450512 (1002182,1898468)	110.97 (76.86,146.11)	3979454 (2759473,5262333)	114.38 (78.55,151.18)	1.74 (1.67,1.81)	0.03 (0.01,0.04)
**YLLs**						
both	9162525 (2183294,24360060)	307.44 (75.01,797.93)	24750579 (6224336,63537378)	309.03 (78.83,786.06)	1.7 (1.54,1.93)	0.01 (-0.04,0.07)
female	6147480 (1495938,15998139)	339.45 (83.24,866.82)	16205906 (4210873,41030981)	342.95 (88.78,870.11)	1.64 (1.45,1.91)	0.01 (-0.05,0.09)
male	3015045 (705164,8361921)	252.03 (59.86,678.28)	8544672 (2045233,23052273)	258.15 (62.51,681.18)	1.83 (1.63,2.12)	0.02 (-0.03,0.1)

**Fig 2:**
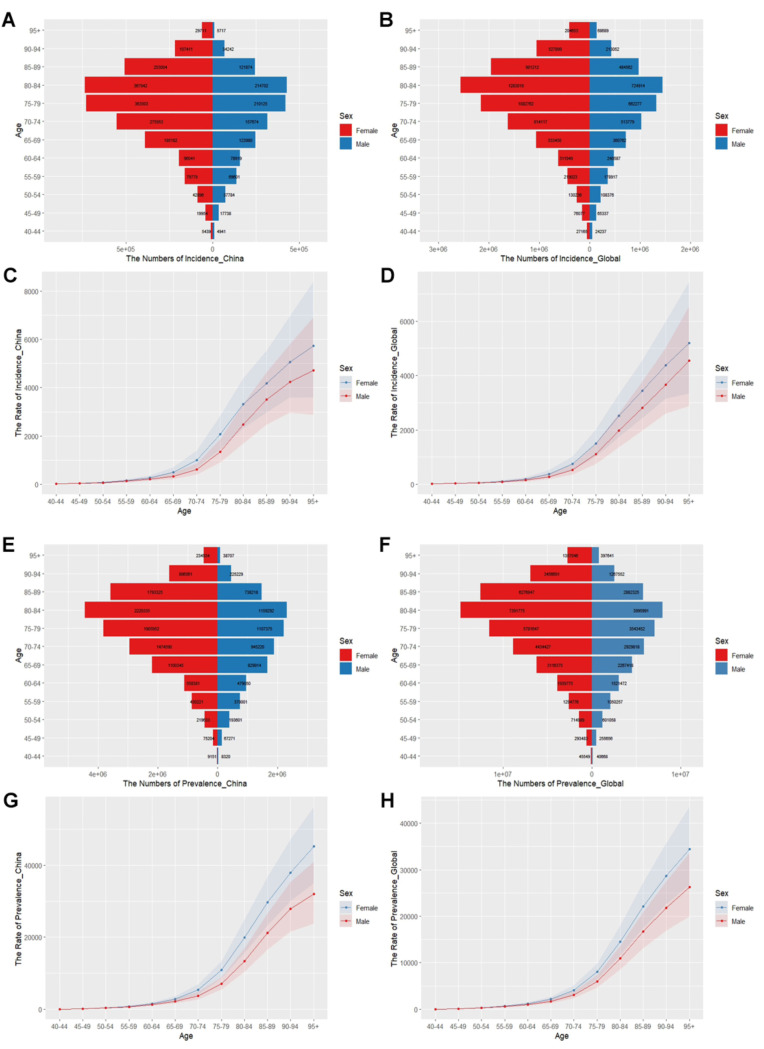
Disease burden of ADD in different age groups and by sex in China and globally in 2021. (A) The numbers of incidence in China (B) The numbers of incidence globally (C) ASIR in China (D) Global ASIR (E) The numbers of prevalence in China (F) The numbers of prevalence globally (G) ASPR in China (H) Global ASPR.

Fig 2 illustrates the burden of disease in different age groups in China and globally, differentiated by sex. Figure contains incidence, prevalence, ASIR and ASPR of ADD in different age groups in 2021. Both in China and the world, the incidence and prevalence of ADD are higher in women than in men. ADD are most common in people aged 80–84 years, with decreasing incidence and prevalence at older or younger ages (Fig 2A, 2B, 2E, 2F). Using the line graph, we can see that the incidence tends to stabilize before the age of 65 years, with the peaks of the incidence and the prevalence starting between the ages of 65–69 years, after which the slope gradually increases with increasing age (Fig 2C, 2D, 2G, 2H).

**Fig 3:**
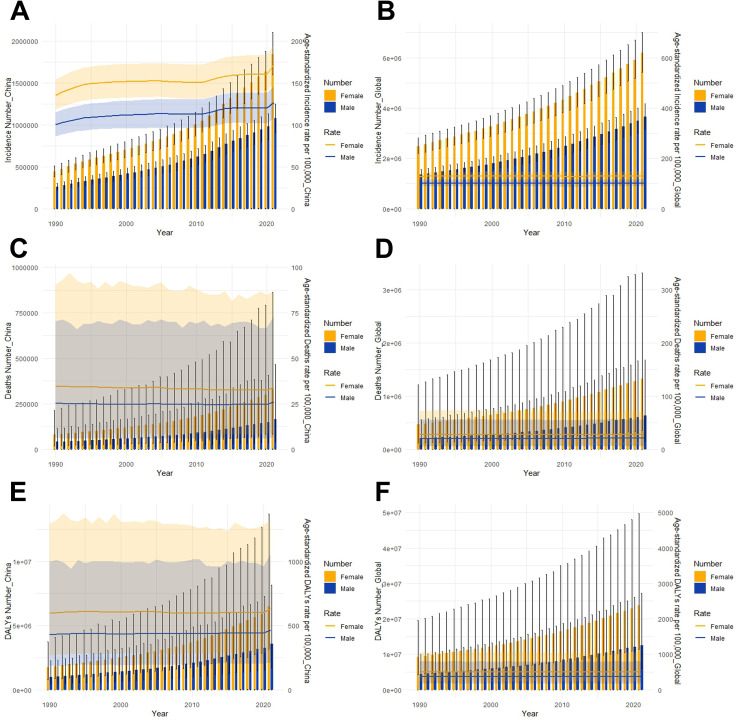
Trends in all-age numbers and ASRs of incidence, deaths and DALYs for ADD by sex, 1990-2021. The numbers on the left correspond to the bar graph, and the rates on the right correspond to the curve graph. (A) Incidence number and rate in China (B) Global incidence number and rate (C) Deaths number and rate in China (D) Global deaths number and rate (E) DALYs number and rate in China (F) Global DALYs number and rate.

Generally speaking, the all-age numbers of incidence, deaths and DALYs for both sexes in China and globally have shown increasing trends of varying degrees (Fig 3). But the trends in ASRs are different. In China, there is an overall increasing trend in ASIR and a mild decreasing trend in ASDR among men and women with ADD (Fig 3A, 3C). The age-standardized DALYs rate stabilizes overall, with a slight upward trend after 2020 (Fig 3E). Compared to the Chinese data, worldwide ASIR, ASDR and age-standardized DALYs rate do not change significantly, which is also consistent with the results obtained in Table 2 (Fig 3B, 3D, 3F).

### Joinpoint regression analysis

**Fig 4:**
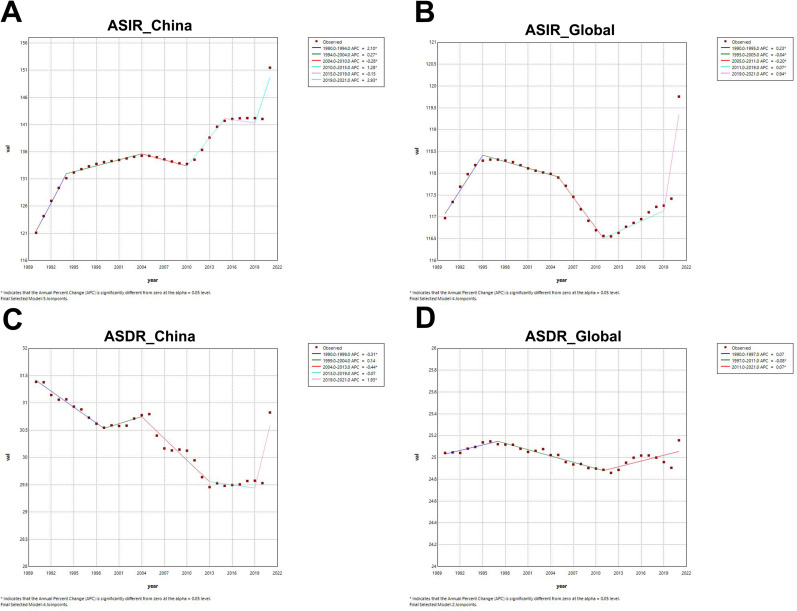
Joinpoint regression analysis of ADD among all sexes in China and Globally, 1990-2021. An asterisk in the upper right corner of an APC value means that the *P*-value for that APC value is less than 0.05. (A) Segmental trend of ASIR in China (B) Segmental trend of global ASIR (C) Segmental trend of ASDR in China (D) Segmental trend of global ASDR.

Using joinpoint regression analysis, we found obvious differences in the segmentation trends of ASIR and ASDR between China and the world. The incidence of ADD in China has been increasing since 1990, and the APC values were statistically significant in every year interval except 2015–2019, with the greatest increases especially in 1990–1994 (APC = 2.10) and 2019–2021 (APC = 2.93) (Fig 4A). In contrast, worldwide incidence shows a wave-like pattern. In Fig 4B, a downward trend in the incidence of ADD can be seen globally over the period 1995–2011 (APC_1995–2005_ = -0.04 and APC_2005–2011_ = -0.20), with a return to an upward trend after 2011. The *P*-value of AAPC for China and the world is less than 0 for the whole period, with China’s AAPC value converging to 1 and the world’s AAPC value converging to 0 (Table 3).

**Table 3:** AAPC values of ASIR and ASDR for ADD in China and globally, 1990-2021.

	Incidence		Deaths	
	AAPC (95% CI)	*P* value	AAPC (95% CI)	*P* value
**China**				
both	0.68 (0.63 - 0.72)	0	-0.09 (-0.17 - 0)	0.049
female	0.71 (0.55 - 0.87)	0	-0.10 (-0.20 - 0)	0.045
male	0.71 (0.66 - 0.76)	0	0.09 (0.01 - 0.17)	0.023
**Global**				
both	0.06 (0.05 - 0.07)	0	0 (-0.02 - 0.03)	0.785
female	0.10 (0.08 - 0.11)	0	0.02 (0 - 0.04)	0.067
male	0.07 (0.05 - 0.10)	0	0.09 (0.05 - 0.11)	0

Unlike ASIR, ASDR in China shows a continually low trend (Fig 4C). The AAPCs of the entire Chinese population and women were negative, but the AAPC of the ASDR of Chinese men is 0.09 (95% CI, 0.01 to 0.17, *P* = 0.023), indicating that their mortality rate was showing a slight upward trend (Table 3). The global ASDR has remained largely stable. As in the case of China, the burden of ASDR worldwide is also greater for men according to gender (Table 3).

### Decomposition analysis

**Fig 5:**
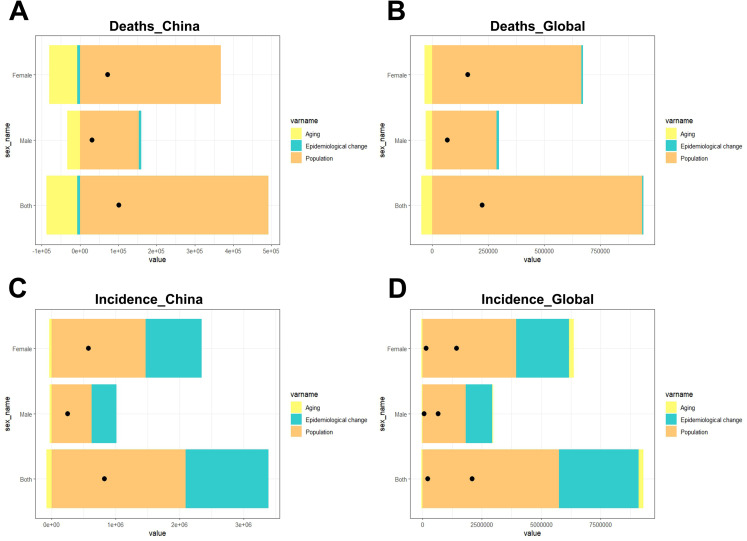
Decomposition analysis of ADD by sex according to aging, population and epidemiological change in China and Globally, 1990-2021. The black dots indicate the overall trend resulting from adding the contributions of the three components. (A) Distribution of causes of deaths in China (B) Global distribution of causes of deaths (C) Distribution of causes of incidence in China (D) Global distribution of causes of incidence.

Between 1990 and 2021, the disease burdens of ADD have increased more for women than for men (Fig 5). Population growth was the most important cause of increased mortality and incidence in China and globally. Aging had a negative impact on mortality in China and the world, as well as on the incidence of ADD in China (Fig 5A, 5B, 5C). While, aging had both negative and positive effects on the global incidence of ADD, and the positive impact was greater (female: 3.4%; male: 0.73%; both: 2.46%) (Fig 5D). The effect of epidemiological changes on the increase in mortality among Chinese women and the Chinese population as a whole was negative (female: -2.63%; both: -1.79%) (Fig 5A), but the effect on the rest of the population distribution was positive. Epidemiological changes accounted for a larger proportion of the increase in incidence than mortality. Because the impacts of epidemiological changes on the increase in mortality were only in the single digits, but the impacts on the increase in incidence were as high as about 40 percent (Table 4).

**Table 4:** Percentage of aging, population and epidemiological change for ADD by sex in China and globally, 1990-2021.

	Incidence			Deaths		
	Aging_ percent (%)	Population_ percent (%)	Epidemiological change_ percent (%)	Aging_ percent (%)	Population_ percent (%)	Epidemiological change_ percent (%)
**China**						
both	-2.46	63.38	39.08	-19.84	121.63	-1.79
female	-1.81	63.87	37.94	-25.32	127.95	-2.63
male	-3	63.15	39.85	-26.07	121.34	4.73
**Global**						
both	2.46	57.48	40.06	-5.33	104.66	0.67
female	3.4	57.51	39.09	-5.4	104.42	0.98
male	0.73	57.65	41.62	-10.74	106.23	4.52

### Risk factors for ADD

**Fig 6:**
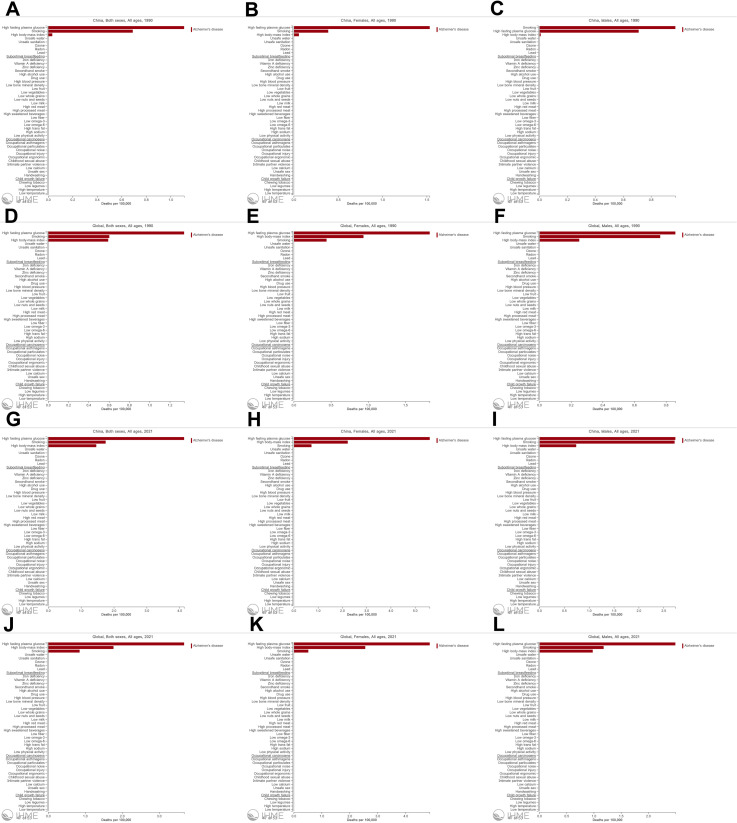
Attributable risk factors for ADD by sex in China and globally, 1990 and 2021. (A) Risk factors for both sexes in China, 1990 (B) Risk factors for females in China, 1990 (C) Risk factors for males in China, 1990 (D) Risk factors for both sexes globally, 1990 (E) Risk factors for females globally, 1990 (F) Risk factors for males globally, 1990 (G) Risk factors for both sexes in China, 2021 (H) Risk factors for females in China, 2021 (I) Risk factors for males in China, 2021 (J) Risk factors for both sexes globally, 2021 (K) Risk factors for females globally, 2021 (L) Risk factors for males globally, 2021.

Attributable risk factors for ADD include smoking, high fasting plasma glucose and high body-mass index. In 1990, the risk factor in most populations was led by high fasting plasma glucose, followed by smoking and high body-mass index (Fig 6A, 6B, 6D, 6E, 6F). However, as shown in Fig 6C, the top risk factor for ADD among Chinese men in 1990 was smoking. By 2021, the top morbidity risk both in China and globally becomes high fasting plasma glucose, in both men and women (Fig 6G, 6H, 6I, 6J, 6K, 6L). In Figs 6F and 6I, we found that the risk of smoking for global men in 1990 and Chinese men in 2021 had converged to high fasting plasma glucose, so we still cannot ignore the risk of smoking for ADD, especially in the male population.

We then estimated all-age numbers and age-standardized rates of DALYs, YLDs, and YLLs for these three risk factors in 2021 and compared the Chinese situation with the global one (Table 5). High fasting plasma glucose is a major contributor to DALYs in ADD in China and worldwide. The age-standardized DALYs rates were 66.72 (95% UI, 3.91 to 177.01) in China and 66.42 (95% UI, 3.83 to 178.85) in the world, which were very similar. There was also consistent with the ranking of attributed risk factors demonstrated by Fig 6.

**Table 5:** All-age numbers and ASR per 100,000 of DALYs, YLDs and YLLs of attributable risk factors for ADD by sex in China and globally in 2021.

		Smoking		High fasting plasma glucose		High body-mass index	
	Measure	All-age numbers (95% UI)	Age-standardized rate per 100,000 (95% UI)	All-age numbers (95% UI)	Age-standardized rate per 100,000 (95% UI)	All-age numbers (95% UI)	Age-standardized rate per 100,000 (95% UI)
**China**	**DALYs**						
	both	602501.08 (257945.38,1379582.96)	30.63 (12.98,69.65)	1204039.42 (70781.95,3184253.24)	66.72 (3.91,177.01)	466728.68 (-36467.55,1800202.16)	24.75 (-1.75,98.65)
	female	105335.58 (42541.44,239663.5)	10.08 (4,23.43)	771929.66 (46281.17,2061089.58)	74.65 (4.46,198.27)	340916.87 (-37073.64,1293098.82)	32.02 (-3.22,123.76)
	male	497165.5 (208749.72,1158493.86)	58.01 (23.81,135.68)	432109.75 (25175.19,1174803.3)	55.42 (3.12,148.24)	125811.81 (-2825.76,489745)	15.28 (-0.27,61.44)
	**YLDs**						
	both	208980.55 (125850.75,310724.45)	10.32 (6.22,15.46)	411880.82 (36863.7,893080.45)	21.99 (1.97,48.14)	159401.84 (-15295.82,511229.01)	8.11 (-0.72,26)
	female	37400.48 (20779.01,58526.29)	3.51 (1.93,5.48)	269353.58 (24003.37,585586.77)	25.53 (2.27,55.98)	119046.13 (-14671.51,383598.04)	10.95 (-1.27,35.44)
	male	171580.06 (105753.14,253792.56)	18.91 (11.69,28.29)	142527.23 (12860.34,311071.93)	17.18 (1.55,37.63)	40355.71 (-1516.58,123317.36)	4.57 (-0.12,14.39)
	**YLLs**						
	both	393520.53 (92582.05,1133291.35)	20.31 (4.76,57.59)	307326.84 (-22653.01,1347774.19)	16.64 (-1.09,75.47)	792158.6 (31686.53,2504933.47)	44.74 (1.77,139.34)
	female	67935.09 (14850.72,189076.55)	6.58 (1.41,18.09)	221870.73 (-20609.34,971001.38)	21.07 (-1.83,92.96)	502576.08 (20080.46,1555882.09)	49.12 (1.94,153.96)
	male	325585.44 (76978.08,962644.79)	39.11 (9.32,115.58)	85456.1 (-1333.43,400264.3)	10.7 (-0.15,50.44)	289582.52 (11704.62,929181.59)	38.24 (1.53,126.68)
**Global**	**DALYs**						
	both	1533213.54 (662722.71,3496419.97)	18.36 (7.9,42.07)	5348853.67 (308059.6,14351156.38)	66.42 (3.83,178.85)	2665746.28 (-494344.95,9332493.37)	32.86 (-5.97,115.18)
	female	423190.66 (185084.81,947536.47)	9.01 (3.94,20.16)	3419724.59 (198317.96,9146429.2)	72.55 (4.21,193.7)	1881162.5 (-377248.21,6557379.42)	39.91 (-8.02,138.91)
	male	1110022.88 (474038.47,2558503.5)	30.56 (12.72,71.5)	1929129.08 (109741.64,5194917.67)	57.71 (3.29,155.61)	784583.78 (-117096.74,2808635.11)	23.18 (-3.28,82.98)
	**YLDs**						
	both	1687335.5 (144491.16,3705524.99)	20.71 (1.78,45.43)	845484.28 (-205930.2,2474059.97)	10.28 (-2.47,30.17)	515882.19 (313106.13,759417.35)	6.12 (3.72,9.03)
	female	1083188.05 (92294.16,2396401.02)	23.07 (1.96,50.98)	601363.51 (-158801.39,1759232.86)	12.82 (-3.39,37.53)	143042.41 (84581.66,215145.63)	3.06 (1.81,4.59)
	male	604147.44 (52197.01,1322693.41)	17.51 (1.52,38.29)	244120.78 (-47008.28,721716.37)	6.94 (-1.27,20.72)	372839.78 (228453.35,545265.06)	9.99 (6.16,14.8)
	**YLLs**						
	both	3661518.17 (148109.57,11503103.12)	45.71 (1.85,143.5)	1017331.35 (241831.62,2876261.39)	22.58 (-3.78,94.44)	1820262 (-309143.68,7598973.29)	12.24 (2.9,34.28)
	female	2336536.53 (95441.38,7266127.24)	49.48 (2.02,153.74)	280148.26 (65583.25,771699.06)	27.1 (-4.86,110.94)	1279799 (-229328.13,5239814.53)	5.95 (1.4,16.41)
	male	1324981.64 (52668.19,4243716.55)	40.21 (1.59,128.9)	737183.1 (177427.4,2115307)	16.24 (-2.14,68.98)	540463 (-75645.66,2352148.32)	20.58 (4.9,58.2)

### Projections of ASDR and ASIR over the next 15 years

**Fig 7:**
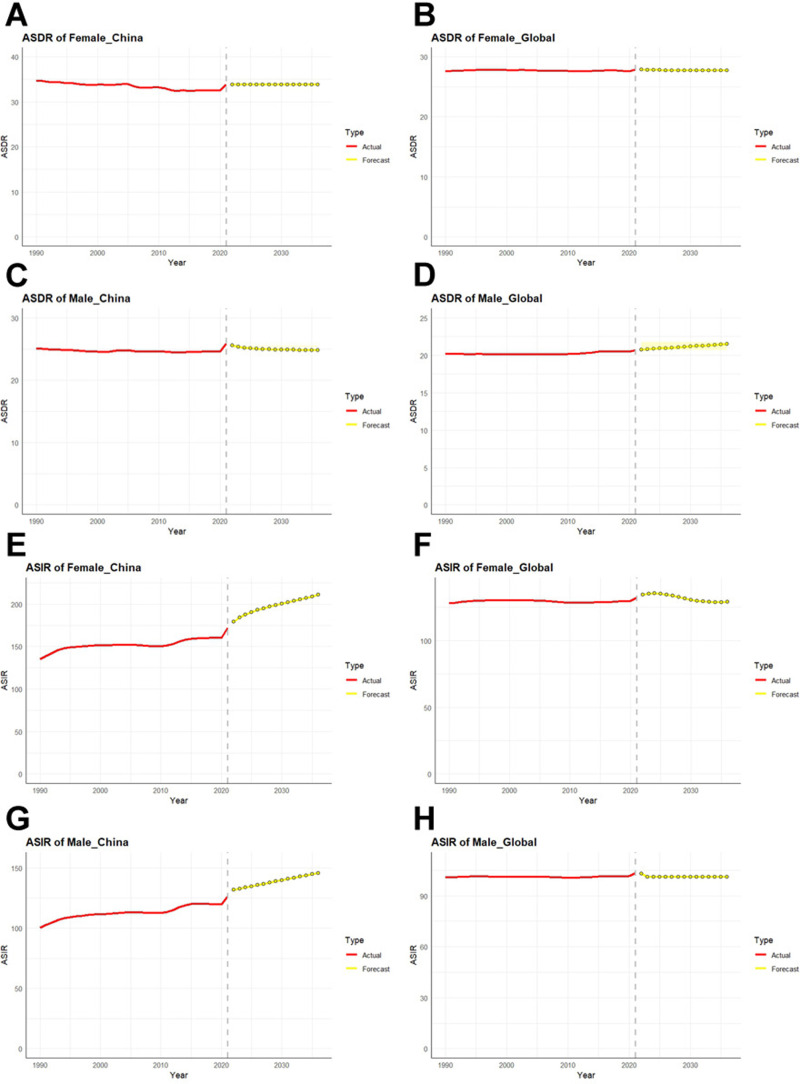
Trend projections of ASDR and ASIR for ADD by sex in China and Globally over the next 15 years. The red line represents the actual trend from 1990 to 2021, while the yellow dotted line and the corresponding shaded area represent the forecast trend and its 95% CI. (A) Prediction of ASDR for female in China (B) Prediction of ASDR for global female (C) Prediction of ASDR for male in China (D) Prediction of ASDR for global male (E) Prediction of ASIR for female in China (F) Prediction of ASIR for global female (G) Prediction of ASIR for male in China (H) Prediction of ASIR for global male.

The ARIMA model was used to predict the incidence and mortality of China and the world in the next 15 years. ASDRs for women in China and worldwide stabilize over the next 15 years and do not show an evident upward or downward trend (Fig 7A, 7B). However, the ASDR trend for males has changed and is in the opposite direction. It can be seen that the ASDR of Chinese males is slightly decreasing, while the one of global males is slowly increasing (Fig 7C, 7D).

In incidence projections, ASIR in men globally is the only burden for which the trend has not changed (Fig 7H). The incidence of ADD in Chinese men and women will increase significantly in the next 15 years, with a higher incidence in women (Fig 7E, 7G). The ASIR for women worldwide will show a small increase in the next 5 years, followed by a leveling off over the next 15 years (Fig 7F, 7G).

## Discussion

### Overview

In an article published in “*The Lancet. Neurology*”, GBD 2021 Nervous System Disorders Collaborators indicated that in 2021, 340 million people suffered from neurological diseases and 11.1 million died from them, posing a significant challenge to public health [20]. This study found that over the past 32 years, there has been a substantial increase in the absolute number of cases for ADD in terms of incidence, prevalence, mortality, DALYs, YLDs, and YLLs. China’s indicator values have increased by three times, while the global values have increased by one time (Tables 1 and 2). The GBD 2016 Dementia Collaborators found that the number of people with ADD worldwide more than doubled from 1990 to 2016 [19]. Despite the addition of five years of data, the global trend in the burden of ADD has not shown significant fluctuations and continues to exhibit a steady growth pattern. The elderly aged 80–84 are the peak population for the incidence and prevalence of ADD, with a rapid increase in ASIR and ASDR after the age of 70, which is similar to the result of the study by Li et al [31].

After removing the effects of aging and population growth, the percentage changes in ASR of various indicators for ADD are all trending towards 0, demonstrating that demographic changes are the main contributors to the increasing burden of the disease (Tables 1 and 2). Numerous past studies have shown that aging leads to a general increase in the incidence and prevalence of ADD, a phenomenon observed in multiple aging countries [5,6,32]. Through the decomposition analysis in this study, we found that population growth is the primary factor contributing to the incidence and mortality of ADD (Fig 5). Aging contributes negatively to both, especially in the analysis of mortality. This may be because as actual lifespan increases, death rates naturally decline. Additionally, epidemiological changes leading to fundamental alterations in population structure have also contributed a small fraction to the incidence of ADD.

In addition, we can also observe that although the percentage changes of ASRs in China are also around the value of 0, the numerical values are larger than those globally, indicating that the increase in ADD disease burden in China is greater than that worldwide (Tables 1 and 2). This is consistent with some previous research findings [33,34]. Our Joinpoint regression analysis also yielded the same results in Fig 4. In China, the ASIR has been increasing annually, while the ASDR has been decreasing annually, with statistically significant AAPC values (*P* < 0.05). Although the global ASIR has shown significant fluctuations over the past 32 years, the overall AAPC value still trends towards 0. The ASDR has not even shown noticeable fluctuations, and the *P-*value of the AAPC is also greater than 0.05.

### Differences in disease burden among ADD of different genders

When categorized by gender, the ASRs for all indicators are highest among females, followed by both sexes and males. Multiple researches have shown that due to differences in hormone levels, brain development, genetic factors, and mental states compared to males, the burden of ADD among females consistently remains higher than that among males [35–37]. In this study, high fasting plasma glucose was the primary attributable risk factor for ADD in the entire population in 2021. Such high metabolic factors undoubtedly lead to a higher burden and risk of ADD for women compared to men, in almost all countries and regions [7,38]. Apart from the risk factors covered in this study, low educational attainment can also contribute to gender-related data disparities [7,39]. Therefore, whether in China or globally, the ASIRs for females are expected to show a pattern of localized or sustained increase, with a higher growth rate than that for males over the next 15 years. In the future, it will be necessary to develop effective intervention measures for female patients with ADD.

Although the disease burden was higher for females than males, our study results showed that the total percentage changes in ASDR, DALYs, and YLLs were higher for males than for females. Even in China, the trends for ASDR and YLLs among males were positive, whereas they were negative among females. This means that females do not lose too many years of life due to premature death. Similarly, Tower J found that women have a longer life expectancy than men [40]. The results of the joinpoint regression analysis also indicated that the AAPCs for deaths were higher among males than females. If not controlled, the ASDR of men worldwide will continue to rise in the future. Numerous information points to the fact that the death burden of male ADD patients cannot be ignored. Smoking was the leading risk factor for ADD among Chinese men in 1990. By 2021, we can observe that although smoking ranks second in terms of risk factors for ADD among men, its risk level is not significantly different from that of high fasting plasma glucose. Smoking can increase the risk of ADD, especially in the male population [41,42].

### Risk factors for ADD

When purely distinguished by gender, women are susceptible to the effects of high metabolism and low educational attainment, while men are prone to the negative effects of smoking. However, we obtained a unified top attributable risk factor when we observe ADD at the national level and the overall population level. The higher the development level of a country, the greater the burden of ADD caused by high fasting plasma glucose will be. As a developing country, China has experienced a significant increase in the number of diabetes patients because of economic transformation and major changes in lifestyle [7,43]. Furthermore, due to uneven regional development, malnutrition is more severe among populations in certain medically underdeveloped areas. Early malnutrition increases the risk of high blood sugar, which is therefore a reason for the continued increase in ADD patients [44]. Many chemical components in tobacco are harmful to the nervous system, and smoking can also indirectly increase the incidence of ADD by increasing the risk of diabetes [38]. Currently, ADD cannot be cured. However, smoking, type 2 diabetes, hypertension, hypercholesterolemia, etc., have been suggested as controllable risk factors for ADD [45]. It would also be beneficial to target gender-specific risk factors in order to achieve clinical and research goals of precise early intervention for different populations.

## Advantages and limitations

Our research is the first to use the disease burden indicators of ADD in China and globally for the years 2020 and 2021. After supplementing with the latest data, we found that the disease burden of ADD is becoming increasingly severe, both in China and globally. The findings of our research can help in developing effective intervention measures in advance, such as controlling smoking among male ADD patients and managing high fasting plasma glucose levels through medication. But definite conclusions still require actual data as the basis.

This study still has several major limitations. First of all, ADD encompasses many types of dementia, with AD being the most common, but also including vascular dementia, Lewy body dementia, and others. The detailed indicators of disease burden and risk factors vary for each subtype. However, it is currently not possible to analyze the GBD for each subtype explicitly because GBD 2021 does not include specific data for them. Besides, our study spans a period of 32 years. During this time, diagnostic criteria, healthcare accessibility, and disease codes for ADD have continuously evolved, potentially introducing heterogeneity. Therefore, the interpretation of our analysis results needs to be considered in conjunction with the actual circumstances. Finally, our research provided specific data at the national level in China. If we could further extract and analyze the incidence, prevalence, and DALYs of ADD in various cities, such studies might become even more valuable.

## Conclusion

In summary, this study reveals the burden of disease and attributable risk factors for ADD in China and globally from 1990 to 2021, and predicts the trend of the development of these diseases in the next 15 years. China’s disease burden is among the highest in the world. Whether in China or around the world, the numbers of incidence, mortality and DALYs of ADD are increasing year by year. The disease burden is higher in women than in men, especially between the ages of 80–84 years. Population growth is a major factor in the increase in morbidity and mortality, and aging plays a negative role in the increase in mortality from ADD. High fasting plasma glucose is a major risk factor for ADD, but for men, the risk of smoking should not be underestimated. The incidence in China is expected to increase in the future, so a comprehensive understanding of the disease burden and risk factors can help develop targeted strategies to prevent dementia and ameliorate the emotional stress on families and the economic burden on society.
